# Ultrasensitivity in the Cofilin Signaling Module: A Mechanism for Tuning T Cell Responses

**DOI:** 10.3389/fimmu.2016.00059

**Published:** 2016-02-19

**Authors:** Rocio Ramirez-Munoz, Patricia Castro-Sánchez, Pedro Roda-Navarro

**Affiliations:** ^1^Department of Microbiology I (Immunology), School of Medicine, Complutense University and ‘12 de Octubre’ Health Research Institute, Madrid, Spain

**Keywords:** ultrasensitivity, bistability, cofilin, Slingshot-1, T cell receptor

## Abstract

Ultrasensitivity allows filtering weak activating signals and responding emphatically to small changes in stronger stimuli. In the presence of positive feedback loops, ultrasensitivity enables the existence of bistability, which convert graded stimuli into switch-like, sometimes irreversible, responses. In this perspective, we discuss mechanisms that can potentially generate a bistable response in the phosphorylation/dephosphorylation monocycle that regulates the activity of cofilin in dynamic actin networks. We pay particular attention to the phosphatase Slingshot-1 (SSH-1), which is involved in a reciprocal regulation and a positive feedback loop for cofilin activation. Based on these signaling properties and experimental evidences, we propose that bistability in the cofilin signaling module might be instrumental in T cell responses to antigenic stimulation. Initially, a switch-like response in the amount of active cofilin as a function of SSH-1 activation might assist in controlling the naïve T cell specificity and sensitivity. Second, high concentrations of active cofilin might endow antigen-experienced T cells with faster and more efficient responses. We discuss the cofilin function in the context of T cell receptor triggering and spatial regulation of plasma membrane signaling molecules.

## Actin Dynamics and Activation of T Cells

Initial signaling events triggered by the T cell receptor (TCR) after the specific engagement of antigenic peptide–MHC complexes (pMHC) occur in dynamic TCR microclusters organized at the periphery of the immunological synapse (IS) (1). TCR microclusters migrate to the center of the IS, where they are endocytosed for signaling downmodulation (2). The actin cytoskeleton is essential for the early signaling and centripetal movement of TCR molecules and integrins that precedes TCR downmodulation (1, 3, 4). TCR early signaling promotes the formation of a dynamic network of filamentous actin (F-actin), which, in turn, mediates the maturation of the IS with the formation of a central and a peripheral supramolecular activation cluster (cSMAC and pSMAC, respectively) (5).

Beyond the function in the initial signaling events and IS maturation, actin dynamics have been suggested to regulate the kinetics of the TCR/pMHC engagement. Experiments based on Förster resonance energy transfer (FRET) in live cells have demonstrated that the affinity of the TCR/pMHC interaction is higher but yet short-lived than previously detected by *in vitro* experiments (6). The actin cytoskeleton was proved to promote a high dissociation rate. These data pose the question about how brief TCR interactions can efficiently activate T cells that are scanning antigen-presenting cells (APCs), which frequently contain low densities of surface antigenic pMHC compared with endogenous pMHC. High affinity and brief interactions might assist the serial-specific engagement of TCR molecules compacted together in surface oligomers, so-called nanoclusters or “protein islands” (6, 7). TCR clustering can also help to keep specificity while raising sensitivity of T cells by ensuring the effective half-life or “confinement time” of a TCR–pMHC interaction as predicted by the rebinding model that was recently proposed (8, 9). Antigen-experienced (Ag-e) T cells exhibit bigger TCR nanoclusters that parallel a lower activation threshold than the observed in naïve T cells (10). Thus, it seems that an avidity-maturation process mediates enhanced responses seen in effector or memory T cells (10, 11). The mechanism regulating the organization of cell surface nanoclusters is nonetheless not known. Interestingly, it has been recently proposed that dynamic short actin filaments promote the formation of surface protein oligomers (12). Thus, in addition to controlling kinetic parameters of the TCR/pMHC engagement and the molecular dynamics during early T cell activation, actin dynamics might also be involved in the spatial and temporal organization of cell surface oligomers of signaling molecules.

## Regulation of Actin Dynamics by Cofilin

**Figure 1:**
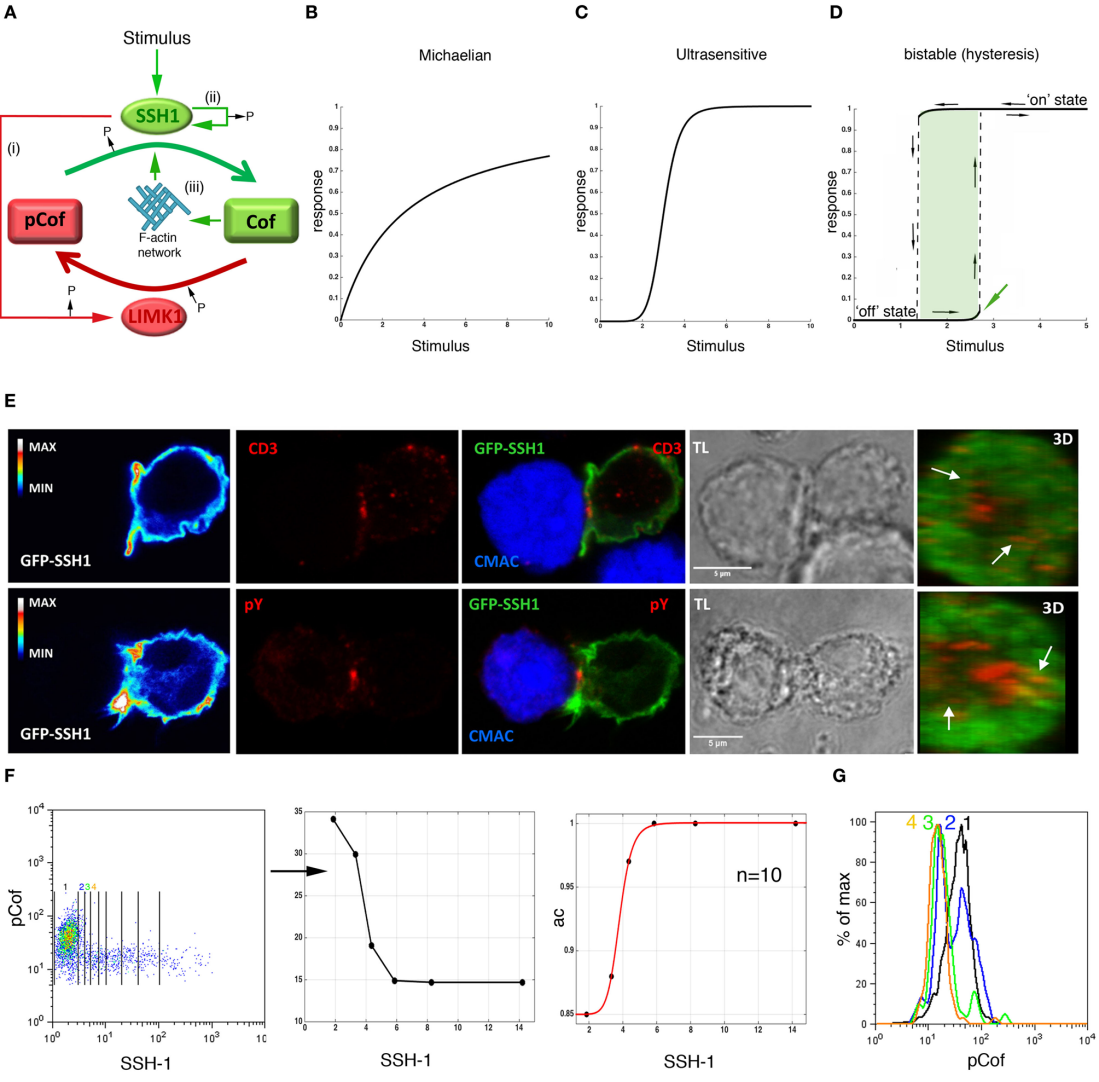
**Ultrasensitivity in the cofilin phosphorylation/dephosphorylation monocycle**. **(A)** Regulation of cofilin activity by SSH-1 and LIMK1. Red and green lines indicate negative and positive regulations, respectively. (i)–(iii) label the reciprocal regulation and positive feedback loops on SSH-1 activation as indicated in the main text. Schematics are shown of graded Michaelian **(B)**, ultrasensitive **(C)**, and bistable **(D)** steady-state response functions. Dashed lines represent intermediate, not possible, states. The green area labels the window of stimulatory inputs generating two stable steady states. A green arrow labels the threshold for switching the module on. Black arrows indicate the going-up and going-down responses characteristic of hysteresis. **(E)** Confocal microscopy of Jurkat CD4 T cells transiently transfected with GFP-SSH-1 and interacting with Raji cells presenting staphylococcal E enterotoxin (SEE). Confocal sections of the green, red, and merged channels as well as three-dimensional (3D) reconstructions of interaction sites are shown. Arrows indicate CD3 clusters and sites of early phosphotyrosine (pY) signaling. Calibration bars quantify the intensity of GFP-SSH-1 (left panels). APCs are identified by staining with 7-amino-4-chloromethylcoumarin (CMAC). TL: transmission light. **(F)** Levels of endogenous phospho-cofilin (pCof) as a function of ectopically expressed GFP-SSH-1 in non-stimulated Jurkat CD4 T cells assessed by flow cytometry (FACS). Lines delimit the regions obtained to plot the mean of pCof versus the mean of GFP-SSH-1 levels (middle panel). Right panel: active cofilin (ac), calculated as [total cofilin(tCof) − pCof]/tCof, as a function of the mean of GFP-SSH-1 levels as before. The tCof was obtained from FACS data (not shown). Black dots are experimental data fitted to a four-parameter Hill equation (red line). Hill exponent (*n*) is indicated. Goodness of fit: SSE 2.2e−6, adjusted *R*-square: 0.9998, and RMSE: 0.00085. **(G)** Histograms of pCof in each of the four regions labeled in **(F)** with color-coded numbers. Note the bimodal distribution of pCof in region 2. Panels show one representative experiment out of three.

Cofilin depolymerizes and severs F-actin, being in this way one of the major regulators of actin dynamics in the cell. Activity of cofilin is regulated by a phosphorylation/dephosphorylation monocycle of the serine residue in position 3 (Ser-3) (Figure 1A). Phosphorylation of Ser-3 by LIM kinases 1 and 2 (LIMK1 and LIMK2) and testicular protein kinases 1 and 2 (TESK1 and TESK2) inactivates cofilin. By contrast, activation of cofilin is mediated by several phosphatases, including serine–threonine phosphatases PP1 and PP2A, chronophin, and a subfamily of dual-specific phosphatases, called Slingshots (SSH-1, SSH-2, and SSH-3) (13). Among Slingshots, SSH-3 does not bind F-actin and shows a less efficient cofilin-phosphatase activity (14). Beyond the regulation by phosphorylation cofilin is also inactivated by PIP_2_ binding at membranes (15) and by oxidative stress conditions (16). Cofilin action on F-actin generates both new barbed ends ready to polymerize and a pool of globular actin to feed polymerization (17–19). In this way, cofilin promotes the formation of a dynamic network of F-actin (20), which is essential for the stimulation of T cells (21). In fact, agents that perturb cofilin dynamics inhibit IS assembly and T cell effector functions (22). Despite the significance of cofilin recruitment to the IS (22), there is no information about the molecular dynamics of cofilin regulators during IS assembly and T cell activation.

## Ultrasensitivity in the Cofilin Signaling Module

Signaling modules based on opposing enzymes, such as the cofilin phosphorylation/dephosphorylation monocycle (Figure 1A), can exhibit different steady-state response functions (Figures 1B–D). When enzymes are working far from saturation and mass action kinetics are assumed, the steady-state response function exhibits a Michaelian shape, which is linear at low stimulatory inputs and tends to a plateau when the amount of substrate decreases with stronger stimulation (Figure 1B). However, properties, such as reciprocal regulations, positive feedback loops, and multiphosphorylation reactions, are known to generate ultrasensitive responses (23), which are characterized by a sigmoidal, switch-like relation between the stimulus and the response, frequently described by the cooperative Hill equation (Figure 1C) (24). In addition to the above-mentioned properties, ultrasensitivity is also generated when substrate levels make both the inhibitory and the activating enzyme to operate close to saturating conditions (so-called zero-order ultrasensitivity) (24), and when a signaling molecule and its activator are concomitantly located to a particular cell compartment (25). Thus, both enzyme levels and molecular dynamics (spatial and temporal regulation) are essential for the output of signaling modules and, consequently, for the cell response. One of the benefits of ultrasensitivity is that it enables cells to filter low stimulatory inputs and to get fast and efficient responses as the stimulus increases. Most importantly, in the presence of positive feedback or double negative feedback loops, ultrasensitivity can also facilitate bistable responses, which constitute real switches in which two stable steady-states are possible (low/“off” and high/“on”) for one particular stimulatory input and an intermediate response cannot take place (Figure 1D) (26). As soon as a threshold is reached, the system turns to the “on” state, where it stays even when the stimulus falls under the threshold level, a property called hysteresis. When positive feedback loops are very strong, bistable responses can be irreversible. In this situation, the “on” state is maintained even when the stimulus is completely depleted. Bistability indicates the existence of a molecular memory controlling the response of the signaling module.

**Figure 2:**
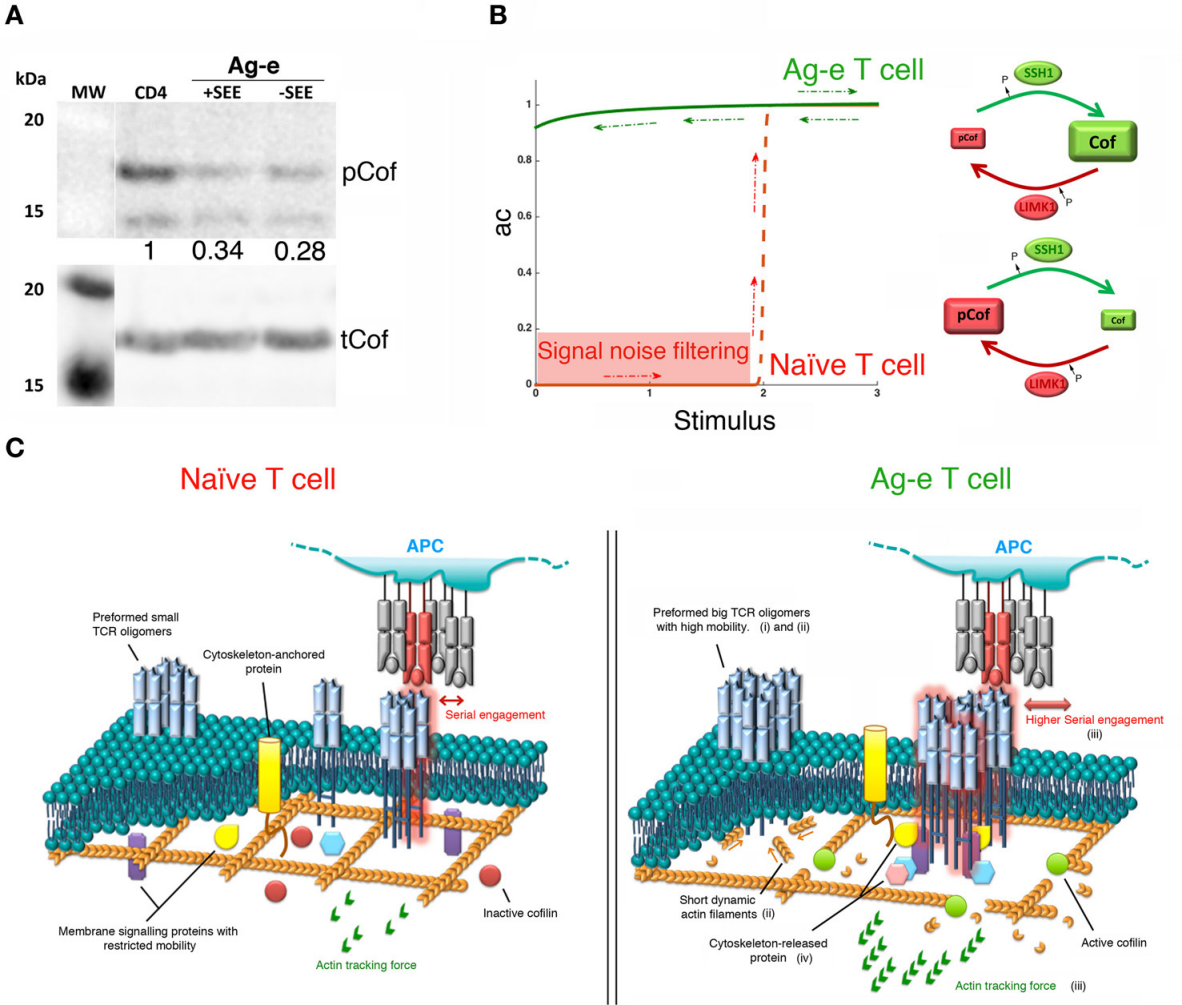
**Bistability in the cofilin signaling module**. **(A)** Western blot showing levels of phospho-cofilin (pCof) and total cofilin (tCof) in resting peripheral blood CD4 T cells and in Ag-e CD4 T cell blasts obtained by stimulating peripheral blood mononuclear cells with staphylococcal E enterotoxin (SEE) for 7 days (labeled as +SEE). In some samples (labeled as −SEE), antigenic stimulation was applied for only 24 h and then washed away leaving cells without the stimulus for 6 days. The western blot of one representative experiment out of five is shown. Numbers indicated the ratio of pCof/tCof normalized to resting cells. **(B)** Left panel: schematic of the proposed steady-state response of active cofilin (ac) as a function of T cell stimulation. The red line shows the ultrasensitive response expected in naïve T cells. Lower stimuli would be filtered out as noise (red area). The green line shows the “on” state of the module proposed for Ag-e T cells. Note, hysteresis (red and green arrows). Right panels: schematics of the cofilin module in naïve and Ag-e T cells. **(C)** Effects of high levels of active cofilin on T cell stimulation in Ag-e cells versus naïve T cells, as explained in the main text by points (i)–(iv).

The cofilin signaling module has several of the above-mentioned properties that generate ultrasensitivity (Figure 1A), including (i) a reciprocal regulation mediated by Slingshot-1 (SSH-1) activation, which activates cofilin and inactivates LIMK1 (27); (ii) a positive feedback loop on SSH-1, which can be self-activated by auto-dephosphorylation (28); and (iii) a positive feedback loop due to the enhanced (1200-fold) cofilin-phosphatase activity of SSH-1 when it is bound to F-actin networks (29), whose organization is promoted by cofilin action (20). These positive feedback loops could promote a bistable response showing hysteresis, especially at high local concentrations of active cofilin that would make LIMK1 to work close to saturation (26). It seems then probable that any stimulus triggering SSH-1 activation will generate an ultrasensitive or bistable response. These regulatory mechanisms of SSH-1 have not been established in either SSH-2 or SSH-3. Supporting a TCR-mediated activation of SSH-1, we found that although an even distribution, with partial colocalization with F-actin, was found in non-stimulated cells (data not shown), GFP-SSH-1 accumulated at peripheral sites in the IS (Figure 1E), as previously described for cofilin (22). These data support the notion that TCR signals could generate an ultrasensitive response of the cofilin signaling module due to the accumulation of cofilin and its activator SSH-1 at the IS. Nonetheless, cofilin-activating signals, such as costimulation (30), might also regulate the dynamics of SSH-1 during T cell activation. It is also plausible that local SSH-1 will be on its highest activation state bound to cortical F-actin. Thus, an efficient inhibition of LIMK1 and, consequently, a reciprocal regulation, is expected at these sites. Consistent with ultrasensitivity, we have found a sharp decrease in the mean of phospho-cofilin amount in T cells as a function of SSH-1 levels (Figure 1F; Figures S1 and S2 in Supplementary Material). Interestingly, the detection at the lowest SSH-1 levels of two discrete populations of active and inactive cofilin with no intermediate states suggests the existence of a bistable response (Figure 1G; Figure S2 in Supplementary Material). In agreement with irreversible bistability and hysteresis, we have found a higher proportion of active cofilin in Ag-e than in resting T cells, even when they were deprived from the antigenic stimulus for 6 days (Figure 2A). In order to further demonstrate irreversibility, it will be needed to design experiments for the complete deprivation of peptide antigenic stimulation by, for example, combining antigen washing and pharmacological inhibition of early TCR signals as previously done (31).

## Physiological Relevance

We propose that the steady-state response of active cofilin as a function of SSH-1 activation may be an irreversible bistable switch (Figure 2B). A fast increase in the actin dynamics is expected once an activation threshold is reached due to TCR and costimulatory signals. Other environmental clues may participate in the regulation of active cofilin during T cell activation. For example, the local reducing environment promoted by dendritic cells at inflammatory sites (32) has been proposed to prevent the inhibition of cofilin activity by PIP_2_ in antigen-specific T cells (33). Interestingly, this might also prevent inhibition of SSH-1 by reactive oxygen species (34). Cofilin ultrasensitive response in coordination with other signaling modules may have potential effects in the sensitivity, specificity, rapidity, and efficiency of switch-like T cell responses.

In naïve T cells, ultrasensitivity might be instrumental in maintaining the peripheral tolerance to low signals emanating from self-peptides while endowing cells with enough sensitivity to foreign antigens. Switching the module to the “on” state will rapidly increase actin dynamics to assist on the early assembly of TCR microclusters after the engagement of antigenic pMHC. This will enable an efficient organization of initial signaling complexes.

Slingshot-1 may participate in the molecular memory that keeps the cofilin signaling module in the “on” state in Ag-e T cells even when the antigenic stimulus is depleted. Although recent findings challenge the notion that Ag-e T cells have lower activation thresholds (35), there is a general agreement about the faster and more efficient responses seen in these cells when compared to their naïve counterparts. High levels of active cofilin will increase the depolymerization and severing of actin. This might promote the following effects in tuning TCR triggering and signaling during the activation of Ag-e T cells (Figure 2C): (i) a higher mobility of TCR nanoclusters. This may raise the chance of finding and engaging antigenic pMHC; (ii) the formation of big TCR oligomers at the cell surface due to abundant short dynamic actin filaments, as described for other surface molecules (12). A bistable response of the cofilin module might then represent a mechanism for the avidity-maturation or for the rebinding model mentioned above; (iii) the generation of stronger actin tracking forces. This would ensure both, more efficient T cell responses by promoting enough short-lived serial engagements of TCR molecules at the larger nanoclusters and the discrimination of low quality ligands; and (iv) a more efficient release of molecules participating in initial events of signaling by the TCR. Recently, it has been shown that TLR signaling in B cells increases the cofilin-dependent actin dynamics and, consequently, reduces the BCR confinement enhancing signaling (36). The same mechanism might also operate in T cells.

In summary, while the cofilin signaling module at the “off” state may guarantee the auto-tolerance and sensitivity of naïve T cells, the “on” state may mediate faster and more efficient responses exerted by Ag-e T cells. Regulation of SSH-1 activity is expected to be essential for switching the cofilin signaling module to the on state. Thus, regulators and dynamics of SSH-1 during the activation of primary T cells should be investigated. A described regulator of SSH-1 during insulin signaling is PI3K (37). PI3K is an effector of ras (38), whose activation is characterized by a bistable response (31). Thus, it seems that T cell responses are tuned by bistability in several signaling modules operating in early steps downstream the TCR (31, 39–41). In order to formally demonstrate the existence of hysteresis, we will need models that enable us to reach maximal stimulation of primary T cells with physiological antigenic peptides and then be able to go down in the stimulation before re-testing the activation state of the signaling module (31). Acute perturbation of signaling components and the cell machinery (such as cytoskeleton and endosomal compartment) should inform about the key players and the dynamics controlling the ultrasensitive response. In this context, dynamic regulation of cofilin by slingshots is under further investigation in our group.

## Author Contributions

RR-M performed experiments, analyzed data, and revised the manuscript. PC-S setup experimental protocols, analyzed data, and revised the manuscript. PR-N contributed to the research design, analyzed data, and wrote the manuscript.

## Conflict of Interest Statement

The authors declare that the research was conducted in the absence of any commercial or financial relationships that could be construed as a potential conflict of interest.

## Abbreviations

ac, active cofilin; Ag-e, antigen-experienced; APCs, antigen-presenting cells; F-actin, filamentous actin; pCof, phospho-cofilin; pMHC, peptide–MHC complex; SEE, staphylococcal E enterotoxin; tCof, total cofilin.

## References

[1] Yokosuka T Sakata-Sogawa K Kobayashi W Hiroshima M Hashimoto-Tane A Tokunaga M Newly generated T cell receptor microclusters initiate and sustain T cell activation by recruitment of Zap70 and SLP-76. Nat Immunol (2005) 6:1253–62. 10.1038/ni1272 16273097

[2] Varma R Campi G Yokosuka T Saito T Dustin ML. T cell receptor-proximal signals are sustained in peripheral microclusters and terminated in the central supramolecular activation cluster. Immunity (2006) 25:117–27. 10.1016/j.immuni.2006.04.010 16860761 PMC1626533

[3] Campi G Varma R Dustin ML. Actin and agonist MHC-peptide complex-dependent T cell receptor microclusters as scaffolds for signaling. J Exp Med (2005) 202:1031–6. 10.1084/jem.20051182 16216891 PMC1373686

[4] Kaizuka Y Douglass AD Varma R Dustin ML Vale RD. Mechanisms for segregating T cell receptor and adhesion molecules during immunological synapse formation in Jurkat T cells. Proc Natl Acad Sci U S A (2007) 104:20296–301. 10.1073/pnas.0710258105 18077330 PMC2154425

[5] Billadeau DD Nolz JC Gomez TS. Regulation of T-cell activation by the cytoskeleton. Nat Rev Immunol (2007) 7:131–43. 10.1038/nri2021 17259969

[6] Huppa JB Axmann M Mortelmaier MA Lillemeier BF Newell EW Brameshuber M TCR-peptide-MHC interactions in situ show accelerated kinetics and increased affinity. Nature (2010) 463:963–7. 10.1038/nature08746 20164930 PMC3273423

[7] Lillemeier BF Mortelmaier MA Forstner MB Huppa JB Groves JT Davis MM. TCR and Lat are expressed on separate protein islands on T cell membranes and concatenate during activation. Nat Immunol (2010) 11:90–6. 10.1038/ni.1832 20010844 PMC3273422

[8] Aleksic M Dushek O Zhang H Shenderov E Chen JL Cerundolo V Dependence of T cell antigen recognition on T cell receptor-peptide MHC confinement time. Immunity (2010) 32:163–74. 10.1016/j.immuni.2009.11.013 20137987 PMC2862301

[9] Dushek O van der Merwe PA. An induced rebinding model of antigen discrimination. Trends Immunol (2014) 35:153–8. 10.1016/j.it.2014.02.002 24636916 PMC3989030

[10] Kumar R Ferez M Swamy M Arechaga I Rejas MT Valpuesta JM Increased sensitivity of antigen-experienced T cells through the enrichment of oligomeric T cell receptor complexes. Immunity (2011) 35:375–87. 10.1016/j.immuni.2011.08.010 21903423

[11] Schamel WW Arechaga I Risueno RM van Santen HM Cabezas P Risco C Coexistence of multivalent and monovalent TCRs explains high sensitivity and wide range of response. J Exp Med (2005) 202:493–503. 10.1084/jem.20042155 16087711 PMC2212847

[12] Gowrishankar K Ghosh S Saha S C R Mayor S Rao M. Active remodeling of cortical actin regulates spatiotemporal organization of cell surface molecules. Cell (2012) 149:1353–67. 10.1016/j.cell.2012.05.008 22682254

[13] Mizuno K. Signaling mechanisms and functional roles of cofilin phosphorylation and dephosphorylation. Cell Signal (2013) 25:457–69. 10.1016/j.cellsig.2012.11.001 23153585

[14] Ohta Y Kousaka K Nagata-Ohashi K Ohashi K Muramoto A Shima Y Differential activities, subcellular distribution and tissue expression patterns of three members of Slingshot family phosphatases that dephosphorylate cofilin. Genes Cells (2003) 8:811–24. 10.1046/j.1365-2443.2003.00678.x 14531860

[15] Yonezawa N Nishida E Iida K Yahara I Sakai H. Inhibition of the interactions of cofilin, destrin, and deoxyribonuclease I with actin by phosphoinositides. J Biol Chem (1990) 265:8382–6. 2160454

[16] Klemke M Wabnitz GH Funke F Funk B Kirchgessner H Samstag Y. Oxidation of cofilin mediates T cell hyporesponsiveness under oxidative stress conditions. Immunity (2008) 29:404–13. 10.1016/j.immuni.2008.06.016 18771940

[17] Chan AY Bailly M Zebda N Segall JE Condeelis JS. Role of cofilin in epidermal growth factor-stimulated actin polymerization and lamellipod protrusion. J Cell Biol (2000) 148:531–42. 10.1083/jcb.148.3.531 10662778 PMC2174812

[18] Ichetovkin I Grant W Condeelis J. Cofilin produces newly polymerized actin filaments that are preferred for dendritic nucleation by the Arp2/3 complex. Curr Biol (2002) 12:79–84. 10.1016/S0960-9822(01)00629-7 11790308

[19] Van Troys M Huyck L Leyman S Dhaese S Vandekerkhove J Ampe C. Ins and outs of ADF/cofilin activity and regulation. Eur J Cell Biol (2008) 87:649–67. 10.1016/j.ejcb.2008.04.001 18499298

[20] Ghosh M Song X Mouneimne G Sidani M Lawrence DS Condeelis JS. Cofilin promotes actin polymerization and defines the direction of cell motility. Science (2004) 304:743–6. 10.1126/science.1094561 15118165

[21] Huang Y Burkhardt JK. T-cell-receptor-dependent actin regulatory mechanisms. J Cell Sci (2007) 120:723–30. 10.1242/jcs.000786 17314246

[22] Eibert SM Lee KH Pipkorn R Sester U Wabnitz GH Giese T Cofilin peptide homologs interfere with immunological synapse formation and T cell activation. Proc Natl Acad Sci U S A (2004) 101:1957–62. 10.1073/pnas.0308282100 14762171 PMC357034

[23] Ferrell JE Jr Ha SH Ultrasensitivity part II: multisite phosphorylation, stoichiometric inhibitors, and positive feedback. Trends Biochem Sci (2014) 39:556–69. 10.1016/j.tibs.2014.09.003 25440716 PMC4435807

[24] Ferrell JE Jr Ha SH Ultrasensitivity part I: Michaelian responses and zero-order ultrasensitivity. Trends Biochem Sci (2014) 39:496–503. 10.1016/j.tibs.2014.08.003 25240485 PMC4214216

[25] Ferrell JE Jr. How regulated protein translocation can produce switch-like responses. Trends Biochem Sci (1998) 23:461–5. 10.1016/S0968-0004(98)01316-4 9868363

[26] Ferrell JE Jr Ha SH Ultrasensitivity part III: cascades, bistable switches, and oscillators. Trends Biochem Sci (2014) 39:612–8. 10.1016/j.tibs.2014.10.002 25456048 PMC4254632

[27] Soosairajah J Maiti S Wiggan O Sarmiere P Moussi N Sarcevic B Interplay between components of a novel LIM kinase-slingshot phosphatase complex regulates cofilin. EMBO J (2005) 24:473–86. 10.1038/sj.emboj.7600543 15660133 PMC548651

[28] Maheswaranathan M Gole HK Fernandez I Lassegue B Griendling KK San Martin A. Platelet-derived growth factor (PDGF) regulates Slingshot phosphatase activity via Nox1-dependent auto-dephosphorylation of serine 834 in vascular smooth muscle cells. J Biol Chem (2011) 286:35430–7. 10.1074/jbc.M111.268284 21857021 PMC3195626

[29] Kurita S Watanabe Y Gunji E Ohashi K Mizuno K. Molecular dissection of the mechanisms of substrate recognition and F-actin-mediated activation of cofilin-phosphatase Slingshot-1. J Biol Chem (2008) 283:32542–52. 10.1074/jbc.M804627200 18809681

[30] Samstag Y Eibert SM Klemke M Wabnitz GH. Actin cytoskeletal dynamics in T lymphocyte activation and migration. J Leukoc Biol (2003) 73:30–48. 10.1189/jlb.0602272 12525560

[31] Das J Ho M Zikherman J Govern C Yang M Weiss A Digital signaling and hysteresis characterize ras activation in lymphoid cells. Cell (2009) 136:337–51. 10.1016/j.cell.2008.11.051 19167334 PMC2662698

[32] Angelini G Gardella S Ardy M Ciriolo MR Filomeni G Di Trapani G Antigen-presenting dendritic cells provide the reducing extracellular microenvironment required for T lymphocyte activation. Proc Natl Acad Sci U S A (2002) 99:1491–6. 10.1073/pnas.022630299 11792859 PMC122218

[33] Schulte B John I Simon B Brockmann C Oelmeier SA Jahraus B A reducing milieu renders cofilin insensitive to phosphatidylinositol 4,5-bisphosphate (PIP_2_) inhibition. J Biol Chem (2013) 288:29430–9. 10.1074/jbc.M113.479766 24003227 PMC3795243

[34] Meng TC Fukada T Tonks NK. Reversible oxidation and inactivation of protein tyrosine phosphatases in vivo. Mol Cell (2002) 9:387–99. 10.1016/S1097-2765(02)00445-8 11864611

[35] Huang J Brameshuber M Zeng X Xie J Li QJ Chien YH A single peptide-major histocompatibility complex ligand triggers digital cytokine secretion in CD4(+) T cells. Immunity (2013) 39:846–57. 10.1016/j.immuni.2013.08.036 24120362 PMC3846396

[36] Freeman SA Jaumouille V Choi K Hsu BE Wong HS Abraham L Toll-like receptor ligands sensitize B-cell receptor signalling by reducing actin-dependent spatial confinement of the receptor. Nat Commun (2015) 6:6168 10.1038/ncomms7168 25644899 PMC4327415

[37] Nishita M Wang Y Tomizawa C Suzuki A Niwa R Uemura T Phosphoinositide 3-kinase-mediated activation of cofilin phosphatase Slingshot and its role for insulin-induced membrane protrusion. J Biol Chem (2004) 279:7193–8. 10.1074/jbc.M312591200 14645219

[38] Wabnitz GH Nebl G Klemke M Schroder AJ Samstag Y. Phosphatidylinositol 3-kinase functions as a Ras effector in the signaling cascade that regulates dephosphorylation of the actin-remodeling protein cofilin after costimulation of untransformed human T lymphocytes. J Immunol (2006) 176:1668–74. 10.4049/jimmunol.176.3.1668 16424196

[39] Stefanova I Hemmer B Vergelli M Martin R Biddison WE Germain RN. TCR ligand discrimination is enforced by competing ERK positive and SHP-1 negative feedback pathways. Nat Immunol (2003) 4:248–54. 10.1038/ni895 12577055

[40] Chan C Stark J George AJ. Feedback control of T-cell receptor activation. Proc Biol Sci (2004) 271:931–9. 10.1098/rspb.2003.2587 15255048 PMC1691681

[41] Acuto O Di Bartolo V Michel F. Tailoring T-cell receptor signals by proximal negative feedback mechanisms. Nat Rev Immunol (2008) 8:699–712. 10.1038/nri2397 18728635

