# Supplementary material for: Ultrasensitivity in the Cofilin Signaling Module: A Mechanism for Tuning T Cell Responses
Source: Front Immunol. 2016 Feb 19;7:59. doi: 10.3389/fimmu.2016.00059 (PMC4759566; doi:10.3389/fimmu.2016.00059)
Supplement: Supplementary file 1 [file data_sheet_1.PDF]

## Supplementary Information

**Ultrasensitivity in the cofilin signaling module: a mechanism for tuning T cell responses**

Rocío Ramírez-Muñoz, Patricia Castro-Sánchez and Pedro Roda-Navarro*
Department of Microbiology I (Immunology), School of Medicine, Complutense University and '12 de Octubre' Health Research Institute, Madrid, Spain

## 1.- Material and Methods

*Cell lines*

Jurkat (JK) clones J77 (Niedergang et al., 1997) and CH7C17 (called here CH7) (Hewitt et al., 1992) were used for confocal microscopy and FACS experiments, respectively. JK and Raji B cell lines used in this work were grown in RPMI 1640 culture medium supplemented with 10% fetal calf serum (Sigma Aldrich, USA), 10 mM glutamine, 100 U/mL penicillin, and 100 µg/ml streptomycin (Lonza Group, Switzerland). CH7C17 were grown in the presence of 4 µg/ml puromycin and 0.4 mg/ml hygromycin B to keep expression of transfected HA-specific Vβ3 TCRs (Hewitt et al., 1992).

*Isolation of primary human CD4 T cells and generation of antigen-experienced (Ag-e) cells*

PBMCs were isolated from human buffy coats by a Ficoll gradient and CD4 T cells were purified using the untouched human total CD4 isolation kit (Invitrogen, USA). Ag-e CD4 T cells were generated by plating PBMCs at 1 million/ml onto P24 wells in the presence of SEE (1 µg/ml), adding 50 u/ml of IL-2 after 24 hours, and leaving them to grow for 6 days (named SEE+). In some samples (named -SEE), cells were washed twice with RPMI medium 24 hours after stimulation with SEE and plated on new fresh wells before IL-2 addition. In all samples, CD4 T cells were purified as before after 7 days.

*GFP-SSH1 construct*

XhoI-SSH-1-HindIII PCR product was amplified from cDNA of human peripheral blood mononuclear cells (PBMCs) using the specific primers 'Fw: CCCCTCGAGTTATGGCCCTGGTGACCCTGCAG' and 'Rv: CCCAAGCTTTTAGCTTTTGCTCATCCACGAAGG'. The amplified PCR product was digested using the XhoI and HindIII restriction sites included in the primer sequences and ligated into a pEGFP-C1 backbone (Clontech Laboratories Inc., Japan). The obtained clone was sequenced at the Genomics facility of the Complutense University of Madrid.

*Transfection*

Five million of JK T cells were transfected with 10 µg of pEGFP-C1-SSH1 or pEGFP-C1 plasmids by electroporation (240 V, 975 F) using the Gene Pulser II system (Bio-Rad Laboratories, USA) (CH7), or by nucleofection using Amaxa® Cell Line Nucleofector® Kit V (Lonza Group) and the Amaxa® Nucleofector® II device (Lonza Group) (J77). Transfected cells were left o/n at 37ºC to allow protein expresion.

*Conjugate formation*

Raji B cells were labeled with 10 μM 7-amino-4-chloromethylcoumarin (CMAC; Life Technologies, USA) for 20 minutes at 37ºC, then loaded with Staphylococcal E Enterotoxin (SEE) (Toxin Technologies, USA) by incubation for 30 minutes at 37ºC followed by three washes with complete media. J77 cells were added at a 1:1 ratio. Cells were spun together to favour interactions and then incubated for 20 min on poly-L-lysine-coated coverslips to allow conjugate formation. Samples were fixed with 4% paraformaldehyde (PFA) for 5 min at room temperature and stored at 4ºC until immunofluorescence staining.

*Immunofluorescence staining*

Before staining, fixed samples were blocked using TBS (20 mM Tris-HCl, 150 mM NaCl) containing 5% of blocking reagent (Roche, Switzerland) and 10 μg/ml of human IgG. Samples were then incubated with anti-CD3 supernatant (Dr Sanchez-Madrid's laboratory, Hospital de la Princesa, Madrid, Spain) followed by incubation with donkey anti-mouse-Alexa Fluor 594 (Life Technologies), or permeabilized with 0.1% Triton X100 and incubated with anti-phosphoTyrosine mouse monoclonal antibody (Cell Signaling Technology Inc., USA), followed by incubation with donkey anti-mouse-Alexa Fluor 594 (Life Technologies).

*Confocal microscopy*

Optical sections of fixed samples were acquired using an Olympus FV1200 confocal scanning laser microscope with a 60X/1.35 oil immersion objective. CMAC, GFP (GFP-SSH1) and A594 (secondary antibodies) were excited by 405, 488 and a 594 nm laser lines, respectively. Z-stacks of at least 20 confocal sections every 0.3 μm throughout the whole cell were acquired. 3D reconstructions of images were made using ImageJ (Fiji) software (NIH; USA).

*FACS staining*

Approximately $10^5$ peGFP-C1-SSH1 or peGFP-C1 transfected CH7 cells were fixed with 2% PFA-PBS for 20 min on ice, permeabilized with 0.2% saponin-PBS for 20 min at RT, and stained with anti-pSer3-Cofilin (Cell Signaling Technology Inc.) or anti-total cofilin antibodies (Dr. Bamburg's laboratory, Colorado State University, USA) followed by Allophycocyanin-labeled goat-anti-rabbit immunoglobulins (Thermo Scientific, USA). The same protocol was followed for experiments done with human peripheral blood CD4 T cells.

The average geometrical mean intensities of pCof and GFP-SSH1 were obtained from labeled gates. The active cofilin (ac) levels were calculated as [total cofilin(tCof)-pCof]/tCof and plotted as a function of the mean of SSH1-GFP levels. The steady-state of active cofilin (ac) as a function of GFP-SSH1 was fitted to a four-parameter hill equation as described(Trunnell et al., 2011). Data processing and fitting was implemented with Matlab (MathWorks, inc. USA).

*Western-blot*

Whole-cell lysates of resting or Ag-e CD4 T cells were generated by incubating cell pellets on ice for 30 minutes with RIPA buffer (20 mM Tris.HCl pH 7.5, 150 mM NaCl, 1% NP-40, 0.5% sodium deoxycholate, 0.1% SDS and 10 mM glycerophosphate) with protease inhibitor cocktail (Roche) plus 10 mM NaF, 1 mM PMSF, and 1 mM $Na_3VO_4$. 4x Laemmli buffer was directly added to cell lysates after sonication and protein quantification.

100 ug of protein were run in a 12% agarose gel and transferred to a PVDF membrane (Merck Millipore, Germany). Western blot was done by 4ºC o/n incubation with polyclonal rabbit anti-pSer3-Cofilin antibody and mouse anti-total cofilin followed by Goat-anti-mouse-680 and goat-anti-rabbit-800 secondary antibodies (LI-COR Biosciences, USA). Membranes were revealed using an Odyssey Imaging system (LI-COR Biosciences) and quantified using Image Studio software (LI-COR Biosciences).

## 2.- Supplementary figures

Figure S1

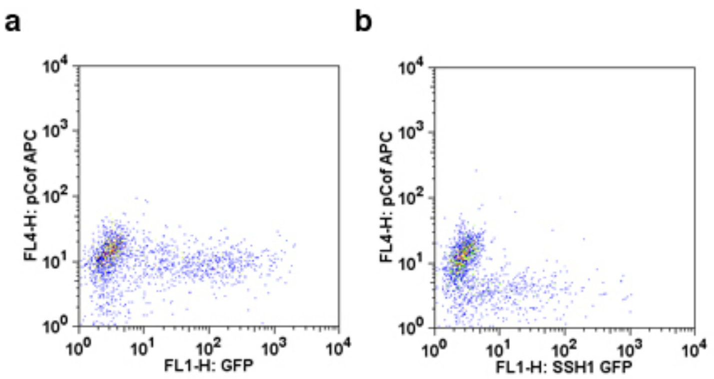


**Figure S1**: pCof expression as a function of GFP (**a**) or GFP-SSH1 (**b**) levels in JK cells. Cells were transfected and stained for pCof as detailed in Material and methods section

Figure S2

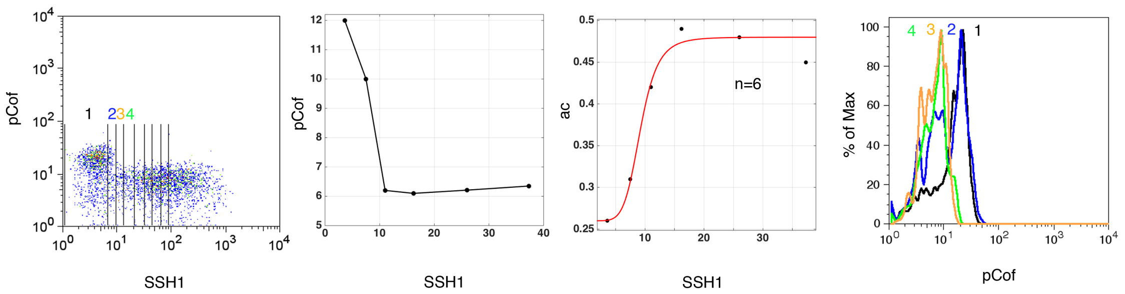


**Figure S2**: pCof expression as a function of GFP-SSH1 levels in human peripheral blood CD4 T cells. The plots, histograms and fit (red line in SSH1/ac plot) were generated as in Figure 1 and explained in material and methods. Goodness of fit: SSE: 0.001198, R-square: 0.9734, Adjusted R-square: 0.9668, RMSE: 0.0173.

## 3.- Bibliography

Hewitt, C.R., Lamb, J.R., Hayball, J., Hill, M., Owen, M.J., and O'Hehir, R.E. (1992). Major histocompatibility complex independent clonal T cell anergy by direct interaction of Staphylococcus aureus enterotoxin B with the T cell antigen receptor. *J Exp Med* 175**,** 1493-1499.

Niedergang, F., Dautry-Varsat, A., and Alcover, A. (1997). Peptide antigen or superantigen-induced down-regulation of TCRs involves both stimulated and unstimulated receptors. *J Immunol* 159**,** 1703-1710.

Trunnell, N.B., Poon, A.C., Kim, S.Y., and Ferrell, J.E., Jr. (2011). Ultrasensitivity in the Regulation of Cdc25C by Cdk1. *Mol Cell* 41**,** 263-274. doi: 10.1016/j.molcel.2011.01.012.
